# Clustering-based methodology for comparing multi-characteristic epidemiological dynamics with application to COVID-19 epidemiology in Europe

**DOI:** 10.1098/rsos.250440

**Published:** 2025-09-24

**Authors:** Alexander Kirpich, Aleksandr Shishkin, Pema Lhewa, Ezekiel Adeniyi, Michael Norris, Gerardo Chowell, Yuriy Gankin, Pavel Skums, Alexander Perez Tchernov

**Affiliations:** ^1^ Department of Population Health Sciences, Georgia State University, Atlanta, GA, United States; ^2^ Department of Computer Science, Georgia State University, Atlanta, GA, United States; ^3^ School of Life Sciences, University of Hawaii at Manoa, Honolulu, HI, United States; ^4^ Quantori, Cambridge, MA, United States; ^5^ School of Computing, University of Connecticut, Storrs, CT, United States; ^6^ Faculty of Mechanics and Mathematics, Belarusian State University, Minsk, Belarus

**Keywords:** clustering-based methodology, COVID-19, epidemiology, Europe, hierarchical clustering, dynamic time wrapping

## Abstract

This study utilized a clustering-based approach to investigate whether countries with similar COVID-19 dynamics also share similar public health and selected sociodemographic factors. The pairwise distances between 42 European countries for six characteristics were calculated, including COVID-19 incidence, mortality, vaccination, SARS-CoV-2 genetic diversity, cross-country mobility and sociodemographic data. Hierarchical clustering trees were constructed, and the strengths of association between the pairs of trees were quantified using cophenetic correlation and Baker’s Gamma correlation measures. The analysis revealed distinct patterns of agreement between clusterings. Vaccination clusterings showed moderate agreement with incidence but no strong agreement with mortality. Mortality-based clustering only agreed with population health clustering. Incidence-based clustering aligned with population health, genetic diversity and selected sociodemographic parameters. Genetic diversity clusterings agreed with mobility and related sociodemographic characteristics. The utility of the cluster-based methods for the time-series is illustrated, and these findings provide insights into the underlying mechanisms driving epidemiological disparities across localities and subpopulations.

## 1. Introduction

The COVID-19 pandemic represents an unprecedented public health crisis, which rapidly spread to the majority of countries worldwide since its first reported case in Wuhan, China [1–5]. Studies have shown considerable variations in COVID-19 incidence, mortality and vaccination coverage across different regions and even within countries in the same region [6–12].

These variations can be attributed to heterogeneous sociodemographic, economic, political and epidemiological factors [10,13]. The list of potential contributing factors is extensive and may include population mobility [14,15], population density [16,17], age structure and life expectancy [18–21], prevalence of cardiovascular diseases, diabetes and obesity [20,21], public health infrastructure [22], gross domestic product (GDP) *per capita* [23,24], human development index (HDI) [25], level of poverty [26], as well as history of spread of SARS-CoV−2 Variants of Concern (VOC) [27–30]. These factors not only directly impacted the spread, morbidity and mortality of the disease but also indirectly influenced the implementation and effectiveness of non-pharmaceutical interventions (NPIs) and vaccination strategies [31–33].

Although there is a vast amount of research on the factors contributing to COVID-19 dynamics, the exact nature of these disparities is not fully understood. Therefore, comprehending the potential root causes of these disparities is vital. This understanding is key to drawing lessons from the COVID-19 pandemic and effectively handling similar health crises in the future.

Simply put, the key question is this: Why did some countries show similar patterns in their COVID-19 epidemiological dynamics, while others differed significantly? A particular aspect of interest is the study of ‘outlier countries’, i.e. those with notably unique public health strategies during the pandemic, such as Sweden and Belarus with their limited NPI policies [34–38] or Italy [39–41], Belarus [37,38] and Russia [42] who reported atypical trends in COVID-19 incidence and mortality. It is crucial to quantitatively assess whether the epidemiological dynamics in these countries significantly deviated from others and, if so, to what extent these differences were due to factors beyond their specific responses to COVID-19.

The study of these questions is distinct from standard association and causation studies. The aim here is not a comparison of incidence and mortality between countries using some metric, but the comparison of *parameters* based on groupings of the studied countries with respect to these parameters. In other words, the major question of this study is not, for instance, ‘Is it the case that a country X and a country Y have similar dynamics of mortality’, but rather ‘Is it generally the case that the countries that express similar dynamics of mortality have also similar dynamics of incidence?’ or ‘Is it generally true that the countries that express similar dynamics of mortality have similar demographic structure?’ Ultimately, the answers to these questions are expected to improve the understanding of the actual trends in incidence and mortality within the given countries. The trends study challenge arises from the need to compare the entire time-series data of incidence, mortality and vaccination rates across countries. These comparisons ideally must consider the *entire time-series* data with dependent records over the entire studied period, rendering regression methods that assume independence between records unsuitable for this analysis. Consequently, straightforward correlation and regression analyses may not be sufficient for studying the similarities in dynamics.

The direct comparison of times series data is widely used for studies of viral epidemics [43–46]. However, COVID-19 time-series data tend to be highly volatile due to multiple reasons, such as variations in public health policies or under-reporting, which vary in time and regionally [47]. To address this inherent variability, several alternative approaches have been proposed. One set of approaches is based on smoothing the time-series data [48], as well as reducing the complete time series to some subset of points, such as doubling time points [49] or turning points [50,51] (e.g. maximums or minimums of epidemic waves). Similar approaches include autoregressive approximation [52] and latent growth model [20]. Other approaches substitute the comparison of entire time series by the comparison of their summary statistics for the entire time period or during particular pandemic waves [52–54].

The above-mentioned techniques help identify general patterns from time-series data and simplify comparisons. However, excessive smoothing and insufficient subsampling could overlook significant region-specific components embedded within specific epidemic sub-waves. Thus, this study utilizes a method of direct pairwise comparison of the complete time-series data from different countries using suitable metrics. These comparisons yield pseudo-distances between the time-series data, which are then utilized as indicators of the similarity between the corresponding countries.

In this paper, a new methodology is introduced which is centred on clustering-based comparative analysis. The goal is to explore potential connections between country similarities in COVID-19 dynamics and similarities by socioeconomic, demographic, geographic and virological factors. European countries were specifically chosen for this study due to the rich diversity, availability and quality of data.

The proposed approach based on clustering has also been established, and a range of methods has already been employed in conducted studies to cluster countries based on COVID-19 data [48,53,55–58]. This manuscript is a continuation and expansion of the clustering approach and the utilization of comparisons between clusterings for different characteristics.

More specifically, the approach proposed in this work is initiated by identifying multiple categories of country characteristics, based on the aforementioned factors or country-specific time series. For each category, a hierarchical clustering tree using specially defined pseudo-distances is generated which measures differences between these characteristics across countries. Then the statistical measures like cophenetic correlation and Baker’s Gamma are utilized for a comparative analysis of these clustering trees within each characteristic. While cophenetic and Baker Gamma measures are not correlations in the classical definition, they are corresponding analogues that characterize the similar relationships between the trees and are therefore also referred to as correlations in the literature. If two characteristics exhibit a high degree of agreement in their clustering patterns, they are considered linked. The use of clustering methods allows to intuitively visualize the groups of countries and see agreements and disagreements between them within the given feature. The hierarchical clustering method was chosen for the analysis since it does not require pre-specifying the number of clusters, allowing potential clusters and similarity patterns to be identified directly from the dendrograms. Furthermore, dendrograms offer intuitive visualizations that are easy to interpret.

It is important to emphasize that the novelty of the proposed approach also lies in its departure from relying on a single clustering solution or a pre-defined number of clusters. Instead, the focus is on comparing multiple clustering outcomes derived from diverse data sources, including the integration of molecular data with classical epidemiological and policy-related data from European countries, which is an additional innovative aspect of the analysis. Also, the proposed method assesses all available variable pairs and does not inherently distinguish between primary variables of interest (e.g. incidence and mortality, in the presented work) and auxiliary explanatory variables (e.g. vaccination rates, mobility or sociodemographic factors). As a result, all variables included in the study are evaluated pairwise, allowing for a direct assessment of how auxiliary variables may influence one another as well as the primary outcomes of interest. Ultimately, this methodology enables (i) the identification of clusters for a given characteristic, which are identified using the most appropriate ‘distance’ metric for that characteristic and (ii) the comparison of those identified clusterings with the corresponding clusterings produced for COVID-19 incidence, mortality and vaccination rates. This insight is instrumental in uncovering the key factors contributing to the variations in COVID-19 dynamics between different European countries.

## 2. Methods

### 2.1. Data collection and prepossessing

This study focused on factors that have already been documented to be associated with the spread of COVID-19 and for which data were available for analysis. Several categories of data were collected, preprocessed and analysed:

(1) COVID-19 surveillance data that include incidence, mortality and vaccination coverage over time.
(2) Full-length SARS-CoV−2 genomes sampled, sequenced and assembled over time.
(3) Pre-pandemic mobility data.
(4) Historic population health data, including life expectancy, cardiovascular disease death rates, diabetes and adult obesity prevalence.
(5) Historic sociodemographic characteristics, including fertility rate, median age, population density, GDP *per capita*, HDI, hospital beds per 1000 individuals and the proportion of individuals living in extreme poverty.

This study focuses on the time period from 22 January 2020 (earliest date available) to 15 February 2022, i.e. before the Russian invasion of Ukraine, which had a severe impact on the epidemiological and data reporting dynamics in Eastern Europe due to massive population movement, disruption of public health systems and COVID-19 surveillance [59–62], thus hampering unbiased comparison of epidemiological and public health data between Eastern European countries and the rest of the continent. Furthermore, so-called micro-states of Andorra, Holy See (Vatican), Liechtenstein, Monaco and San Marino, which are characterized by unusually high testing rates *per capita*, lack of clearly defined borders with their neighbours, and non-typical economics and population structures were excluded from the analysis to avoid skewed results.

COVID-19 incidence and mortality time series were obtained using the data from the COVID-19 Data Repository by the Center for Systems Science and Engineering at Johns Hopkins University [63]. Vaccination data from 21 December 2020 (earliest available) to 15 February 2022 were downloaded from the Bloomberg Vaccine Tracker [64,65]. Cumulative counts were transformed into daily counts, the discrepancies in reporting were addressed by interpolating missing values and, subsequently, by aggregating the data into weekly counts to mitigate reporting effects, such as weekends and holidays. The obtained weekly time series were standardized to the values per population of 100 000, with country populations reported by the UN for the beginning of 2020 [66].

The pre-pandemic inter-country travel data for 2011−2016 (latest processed and available for pre-pandemic period) were obtained from the European Commission Knowledge Center on Migration and Demography [67,68]. The available data did not include Serbia and Montenegro. Consequently, comparisons incorporating mobility data were conducted for 42 countries instead of the original 44 countries. The population health and sociodemographic characteristics of the countries were downloaded from the public repository ourworldindata.org [69], which is a project of the Global Change Data Lab [70], a registered charity in England and Wales.

Finally, genomic data used in this study were obtained from the GISAID repository [71]. This study focuses on comparing time series reflecting distributions of prevalences of major SARS-CoV−2 lineages in analysed European countries. In total, 7,694,400 SARS-CoV−2 sequences sampled over the analysed period were considered. Each sequence’s sampling time, country of sampling and lineage according to the PANGO classification [72] were directly extracted from the GISAID metadata.

### 2.2. Data analysis

The analysis was performed in the following three steps, which are outlined below:

In the first step, the computations of pairwise distances or pseudo-distances between countries for different data types using appropriate metrics (details in the next subsection). As a result, a symmetric 44×44 distance matrix was obtained for every data category (42×42 distance matrix for mobility data). Subsequently, each matrix was standardized by normalizing it by its maximum element.

In the second step, the incidence and mortality distance matrices, which served as the primary data of interest, were visualized using the multidimensional scaling (MDS) method [73–75] as a preliminary analysis to generate hypotheses regarding potential similarities and dissimilarities in terms of the impact of COVID-19 reported incidence and mortality. The primary goal of the MDS method is to perform exploratory analysis, accompanied by visualizations, to generate hypotheses about potential clustering of countries based on reported incidence and mortality. The temporal dimension is incorporated into the distances used in the MDS through the corresponding distance measures for incidence and mortality, as described in the next subsection. The MDS is applied solely to incidence and mortality data, serving as a basis for generating hypotheses to be examined using auxiliary data. This approach, however, subsequently allows for generating hypotheses and further looking into additional characteristics to explore similarities within clusters. Subsequently, the matrices for *each* data category and distance metric were used to perform the hierarchical clustering analysis [76–78] and to produce the hierarchical clustering trees. The hierarchical clusterings were performed using the ‘average’ clustering rule [79,80]. Hierarchical clustering was performed separately for each data type to produce a corresponding complete tree that included all countries in a single hierarchy. This approach enabled direct comparison of the resulting data-specific trees, allowing for the identification of similarities between the hierarchies using appropriate methods. Unlike other methods, hierarchical clustering does not require pre-specifying the number of clusters; instead, it derives the clustering structure during the algorithm’s execution, based on the specified clustering rule. Among different agglomeration methods for hierarchical clustering tree construction, average linkage was selected based on its ability to better preserve the original pairwise dissimilarities among observations, as indicated by the correlation between distances in the resulting tree and the original input distances (median and mean correlation = 0.73 and 0.75 across datasets, respectively).

In the third step, quantification of agreement between hierarchical clusterings trees produced at a previous stage by different categories of data was performed. It was done by comparing hierarchical clustering trees using several metrics, including cophenetic correlation [81,82] and Baker’s Gamma correlation [83,84]. The two separate comparison methods (cophenetic and Baker) were used since they are based on different analysis approaches, allowing for the evaluation of the potential robustness of the results. The summaries of these comparisons were visualized using correlation plots [85] and corresponding graphs [86]. Here, it should be noted that both cophenetic correlation and Baker’s Gamma statistics assess only the structural concordance between the available dendrograms produced earlier, whereas the quality of the clustering and the dendrogram structure itself are determined solely by the hierarchical clustering algorithm applied in the preceding step.

#### 2.2.1. Distance measures for different data types

The distance measures between countries for the *first step* of the analysis were selected based on the data category. For COVID-19 incidence, mortality and vaccinations data, the differences between pairs of countries were computed as the differences between the corresponding time series. Two distinct metrics were used to compute such differences. The first metric was based on the cross-correlation between the time series. Specifically, the cross-correlation distance (CCD) for series X=X(t) and Y=Y(t) was computed as:


$$CCD(X,Y)=1-\max_{l\in L}\left[CC(X,Y,l)\right],$$

where CC(X,Y,l) is the value of the cross-correlation for series X and Y computed for a given value of the lag l. The maximum cross-correlation value was taken across the set of lags L [87]. The CCD(X,Y) formula ensured that countries with the largest cross-correlation values resulted in the smallest values of CCD(X,Y). The second metric was based on the dynamic time wrapping (DTW) algorithm. DTW is a technique used to compare pairs of time series in terms of the distance between them while accounting for their temporal alignment. Specifically, the DTW algorithm identifies the optimal match between two time series in terms of the Euclidean distance based on a defined set of constraints. The corresponding distance between the series is determined from this match [88–90]. In particular, the same CCD and DTW distance measures used to compute distances for incidence and mortality separately *between countries* were also used to compute the distances between incidence and mortality *within every country*. The two methods (CCD and DTW) were considered for comparison purposes since the first method uses standardized correlation values and can be used for series with potentially different scales, while the second method directly relies on the fact that two series should be on the same scale.

The pre-pandemic mobility data were computed as an average mobility during the years 2011−2016. For each pair of countries, the measure of mobility between them was calculated as a total travel count between the countries (in both directions) normalized by their combined population size. The mobility, a similarity measure, was transformed to the distance measure using an exponential transformation. Specifically, the formula used for the mobility pseudo-distance was


$$d(\tau_{ij})=\exp\left[\;-(\tau_{ij}-\tau_{min})/\beta \;\right],$$

where $\tau_{ij}$ represents the standardized travel counts between countries i and j, $\tau_{min}$ is the smallest standardized travel counts across all pairs of countries and β is the mean of all pairwise distances. Additionally, the diagonal values of the mobility pseudo-distance matrix were set to zero.

The remaining sociodemographic and public health data were combined in three different groups. The *population health* characteristics included life expectancy, cardiovascular disease death rate, diabetes prevalence and share of obese adults. The *sociodemographic* characteristics included total fertility rate, median age, population density, GDP *per capita*, HDI, number of hospital beds per 1000 individuals in population and share (proportion) of extreme poverty population. The third group was formed as the combination of the first two groups and included *population health and sociodemographic* characteristics. The values for each considered characteristic were standardized by subtracting the mean of that characteristic across all countries and dividing by the corresponding standard deviation. This resulted in standardized z-scores for each characteristic across countries. As a result, a vector of z-scores was produced for each country based on those characteristics. Those vectors were interpreted as coordinates of each country in multidimensional space and used to compute the Euclidean distance between each pair of countries.

To calculate distances between time series representing distributions of prevalences of major SARS-CoV−2 lineages in analysed European countries, the sequences sampled between 1 April 2020 and 24 February 2022 were considered. The time period was split into T=23 intervals of uniform lengths of 30 days. For each country c, a time series $p^{c}=\left(p_{1}^{c},...,p_{T}^{c}\right)$ was considered, where $p_{t}^{c}=\left(p_{t}^{c}(1),...,p_{t}^{c}(6)\right)$ is the estimated frequency distribution of six SARS-CoV−2 genomic variant options sampled over the time interval t. The variants in question were five VOCs designated by WHO (Alpha, Beta, Gamma, Delta and Omicron), and all genomes not classified as VOCs. The strain distribution distance $SD(c_{i},c_{j})$ between the series $p^{c_{i}}$ and $p^{c_{j}}$ was calculated as the squared differences between distributions $p_{t}^{c_{i}}$ and $p_{t}^{c_{j}}$ averaged over time intervals:


$$SD(c_{i},c_{j})=\frac{1}{T}\sum_{t=1}^{T}\sum_{v=1}^{6}(p_{t}^{c_{i}}(v)-p_{t}^{c_{j}}(v){)}^{2}.$$

The analysis was performed in R [91] and in MATLAB [92]. In particular, the comparison of trees was performed using methods from R package dendextended [93,94]. The entire analysis source code in R and in MATLAB has also been made publicly available on GitHub [95].

The validity and stability of the findings were assessed using a leave-five-countries-out cross-validation approach on a dataset comprising 42 countries. The analysis was repeated 1000 times, each using a randomly selected subset of 37 countries. For each iteration, cophenetic and Baker’s Gamma estimates were calculated. Median values across the 1000 runs, along with 95% confidence intervals defined by the 2.5th and 97.5th percentiles, were reported. This cross-validation method also evaluated the robustness of the results in the presence of potential outliers.

Additionally, the sensitivity of the findings to the length of the aggregation interval was evaluated. While the original analysis was conducted using a 1-week interval, the same procedures were repeated using new aggregation intervals of 2 weeks (a twofold increase in length) and 4 weeks (a fourfold increase in length). The results from these aggregations were then compared.

## 3. Results

### 3.1. Raw data summaries

The standardized weekly raw data summaries for incidence and mortality confirm the high heterogeneity between countries *both* in terms of the scale of detected infections and death *per capita* and the timings of the peaks. Those visual summaries are provided for references in electronic supplementary material, figures S8 and S9.

**Figure 1.**
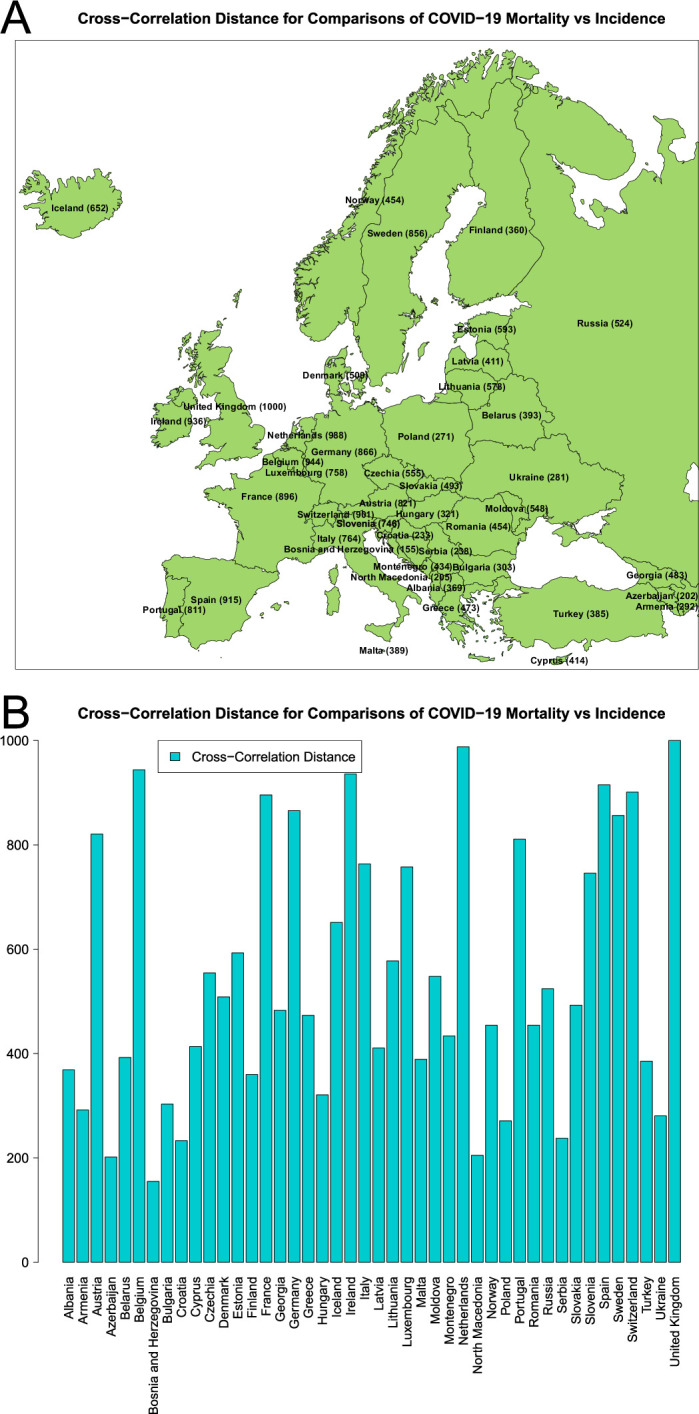
CCD distances standardized on 0–1000 scale between COVID-19 incidence and mortality time series for each of the 44 countries with (A) rounded to integer values summarized on the map and (B) exact values sorted alphabetically. The shapefile used to produce panel (A) has been downloaded from Esri’s hub.arcgis.com and processed in Esri ArcMap v10.7 and Inkscape v0.92.

The more informative summaries of the relationships between incidence and mortality within each country for the entire time series have been computed using CCD and DTW measures. Those CCD and DTW distances between time series were used to compare the continuously evolving dynamics of the relationship between incidence and mortality for the entire studied period. In figure 1, the comparisons utilizing the CCD metric are presented on a standardized 0–1000 scale; see electronic supplementary material, figure S10 for similar plots for the DTW metric. In the above-mentioned figures, the distances between incidence and mortality are provided in two ways for convenience purposes, i.e. rounded to integer values summarized on the map (panel A) and by the distances between the corresponding time series (panel B).

In summary, figure 1 and electronic supplementary material, figure S10 suggest that, although there is a certain correspondence between the overall relative mortality and the distances between incidence and mortality time series, these measures reflect different underlying phenomena. For instance, the countries that exhibit the highest relative mortality, such as Bosnia and Herzegovina, Bulgaria, and North Macedonia, closely align with those that have the smallest incidence/mortality CCD distance, specifically Bosnia and Herzegovina, Azerbaijan and North Macedonia. Yet, the trios of countries exhibiting the most negligible relative mortality and most significant CCD distances, such as Iceland, Norway and Denmark versus Belgium, Netherlands and the United Kingdom, respectively, are markedly distinct. In particular, the ranking of countries based on the distance between their incidence and mortality (as seen in figure 1) aligns with the ranking by the extent of under-reporting of COVID-19 deaths [96].

### 3.2. Multidimensional scaling

**Figure 2.**
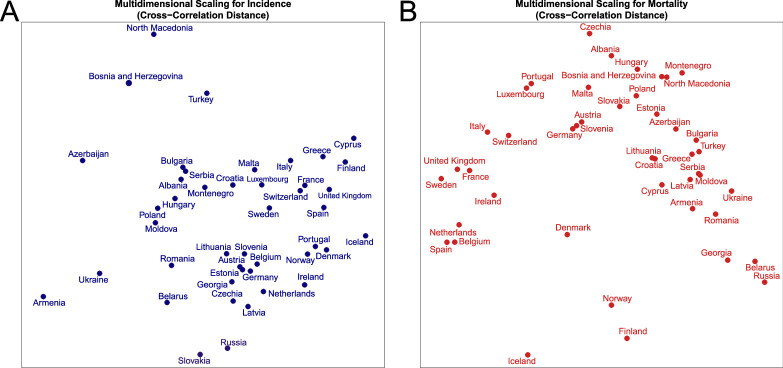
Multidimensional scaling of the CCD distances for (A) incidence and (B) mortality time series of 44 countries.

The MDS provides the preliminary visualizations of dissimilarities between the countries based on the analysed metrics and pseudometrics. In particular, those visualizations can help to identify potential outliers. The visual summaries for CCD and DTW distances between incidence and mortality series are presented in figure 2 and electronic supplementary material, figure S11, respectively. Based on the CCD method for incidence, Armenia, Ukraine, Azerbaijan, Bosnia and Herzegovina, North Macedonia, Turkey, Belarus, Slovakia, Russia and Iceland emerge as the most ‘distant’ from the others, as demonstrated in figure 2A. Most of these countries are in Eastern and Southern Europe, with half belonging to the former Soviet region and 30% to the Balkan region, including Turkey. These associations are statistically significant, with p=0.018 (hypergeometric test [97]) for random sampling of 5 out of 9 post-Soviet countries among 10 outliers and p=0.011 (hypergeometric test) for random sampling of 9 out of 24 Eastern European countries among 10 outliers.

Various factors may explain their ‘outlier’ status in terms of incidence dynamics, such as their unique implementation of NPIs, the population’s adherence to these measures or the potential under-reporting of COVID-19 cases [37,38,98–102]. An exception within this list is Iceland, which does not fall within the previously mentioned regions. However, it may be seen as the exception that validates the rule, given its unique COVID-19 experience, largely due to its geographical isolation and extensive range of public health measures [103–105]. In contrast, Western and Northern European countries cluster more closely, indicating more uniform incidence dynamics.

For mortality (figure 2B), Belarus, Russia, Georgia (former Soviet Union) and Iceland, Finland, Norway and Denmark [106] (Nordic countries) appear to be the ‘outliers’. The appearance of 4 out of 5 Nordic countries among 7 outliers is statistically significant (p=0.001, hypergeometric test). In comparison, for the selection of 3 out of 9 post-Soviet countries among 7 outliers, the p-value was estimated to be 0.11. It is worth noting that the only Nordic country missing from this list is Sweden, which is notable for limited NPI policy during the COVID-19 pandemic [34–36], with indications of high mortality rates. Regarding mortality dynamics, Sweden aligns more closely with Western European countries than its sociocultural and geographic region. Belarus, another European nation that adopted a similar NPI policy [37,38], indeed can be considered as an ‘outlier’, although not by itself but as a part of a small regional cluster. On the other hand, Denmark, whose NPI policies were highlighted in some studies for effectively minimizing both economic costs and the number of deaths [106], also stands out as a mortality outlier. The results were comparable between CCD and DTW methods with some differences.

### 3.3. Comparisons of clusterings for different data types and sources

**Figure 3.**
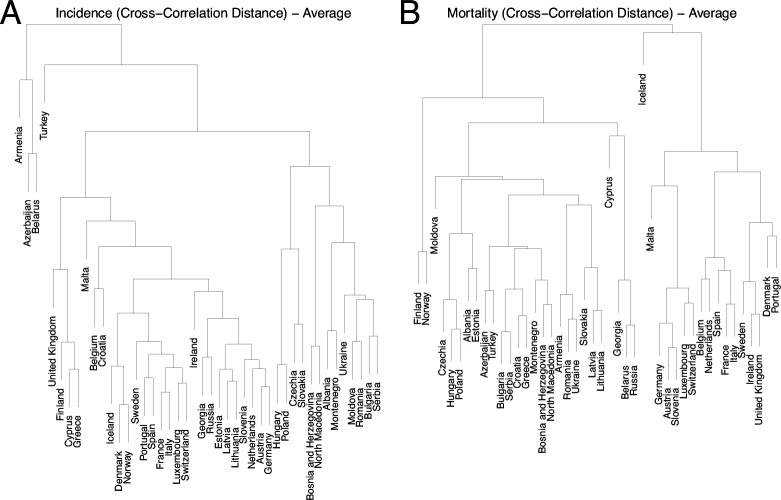
Hierarchical clustering tree for the CCD distance between the 44 countries based on (A) incidence and (B) mortality time series.

Hierarchical clustering trees were constructed and analysed to examine the associations between various epidemiological and sociodemographic factors. These trees were built upon distances or pseudo-distances between analysed country characteristics. For example, figure 3 and electronic supplementary material, figure S12 illustrate the trees for CCD and DTW distances between the incidence and mortality time series for 44 countries. Those trees complement the knowledge gained from MDS in figure 2 by providing precise country clustering based on the tree structure, as opposed to the two-dimensional preliminary visualization of incidence and mortality proximities which MDS utilizes. In the same way, the pre-pandemic average mobility data trees are depicted in electronic Supplementary material, figure S13 for 42 countries (excluding Serbia and Montenegro). Additionally, the trees for population health characteristics, sociodemographic characteristics, the full set of available characteristics and strain distribution distances can be found in electronic supplementary material, Figure S14–S17. Lastly, the trees related to vaccinations are reported in electronic Supplementary material, figures S18 and S19.

Generally, the country clusters based on incidence appear intuitive, as countries that share geographical, economic, cultural or mobility-related ties often group together. This can be seen in Luxembourg and Switzerland, Spain and Portugal, Czechia and Slovakia, Cyprus and Greece, Estonia, Latvia and Lithuania. However, such correlations are not universal, and geographical or cultural groupings do not fully account for the diverse epidemiological dynamics. Notably, clusters based on mortality, vaccination and strain distribution are markedly diverse and deviate significantly from those based on incidence, while the resulting groupings appear less straightforward. Furthermore, the clustering of time series using CCD and DTW methods does not agree perfectly, which may be attributed to the fact that the CCD and DTW metrics rely on different underlying methods, which utilize either cross-correlations or temporal alignment to minimize the Euclidean distance between the time series using a set of constraints.

**Figure 4.**
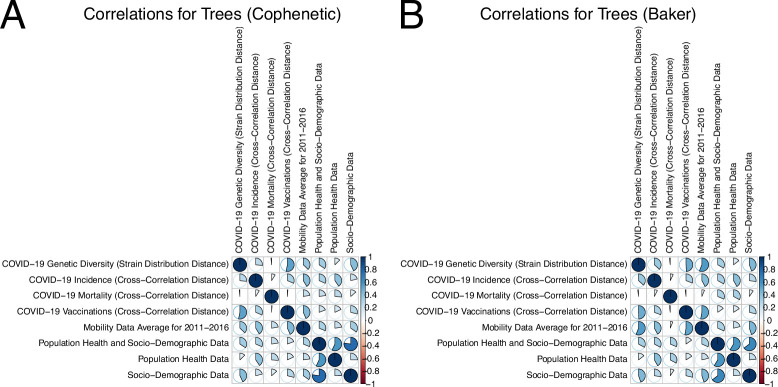
The pairwise summaries between the hierarchical clustering trees for different data sources using the CCD metric for time-series comparison. (A) Cophenetic correlations. (B) Baker's Gamma statistics.

Pairwise measures of agreement can be visually compared in a single figure. Specifically, colour codes based on the values of the measures for all pairs of characteristics can be utilized for this purpose. More specifically, figure 4 and electronic supplementary material, figure S20 summarize the quantitative measures of agreement between hierarchical clustering trees for various characteristics, using CCD and DTW metrics, respectively, for time-series comparison. The comparison measures employed here are cophenetic correlation and Baker’s Gamma, both correlation-based and ranging from −1 to 1, with higher values signifying better agreement. The agreements between the cophenetic correlations and the corresponding Baker’s Gamma statistics values have been evaluated as single-valued Pearson correlation estimates. The estimates were computed between the two tables of values, pairwise for each cell, with Pearson correlation estimates of 0.97 (95%CI:[0.96;0.98]) for CCD and 0.99 (95% CI:[0.98;0.99]) for DTW, respectively. The precise differences for each pair of trees *between* the cophenetic correlations and the corresponding Baker’s Gamma statistics have also been summarized in electronic supplementary material, table S5 and figure S21 for CCD and in electronic supplementary material, table S6 and figure S23 for DTW statistics, respectively. The cophenetic correlation and Baker’s Gamma methods agree fairly well, with some minor discrepancies, most pronounced for Mobility Data Average for 2011−2016. Such minor differences in results have been expected, since cophenetic correlation relies on the standard correlation of the cophenetic distances obtained from each tree, while Baker’s Gamma statistics utilize trees paired in multiple ways and evaluate clusters.

**Figure 5.**
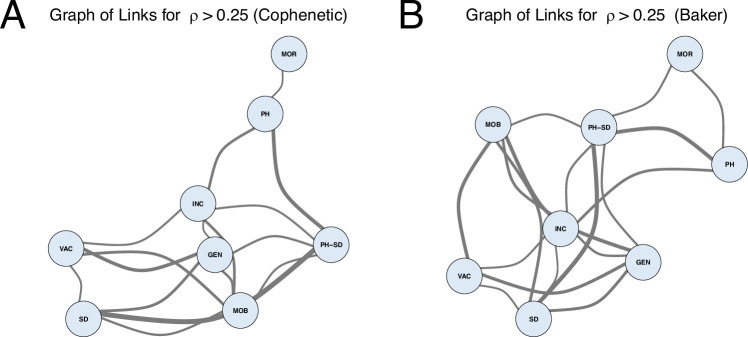
The graph of associations between country characteristics. The vertices represent characteristics and the edges correspond to strong correlations (exceeding the threshold of 0.25). The width of an edge represents the corresponding correlation value. The graph layouts were obtained using Kamada–Kawai algorithm. The short names of the graph vertices have the following meaning: GEN - COVID − 19 Genetic Diversity (Strain Distribution Distance), INC - COVID − 19 Incidence (Cross-Correlation Distance), MOB - mobility Data, MOR - COVID − 19 Mortality (Cross-Correlation Distance), PH - population health data, SD - sociodemographic data, PH-SD - population health and sociodemographic data, VAC - COVID − 19 Vaccinations (Cross-Correlation Distance)

The summaries for both measures are provided to ascertain the robustness of the results. The trees under comparison were developed for 42 countries, excluding Serbia and Montenegro due to the unavailability of their mobility data. For the alternative and potentially more focused visualization of the highest correlation values and interconnectedness between different data sources, the deduced associations between characteristics are also visualized using the corresponding graphs. In particular, figure 4 for CCD and electronic supplementary material, figure S20 for DTW serve as summaries of the entire set of characteristics; they present all pairwise statistics and the relationships, which can be either positive or negative. Moreover, each row of figure 4 for CCD and electronic supplementary material, figure S22 for DTW emphasizes how the corresponding characteristic is related to all other characteristics, providing a general idea of the agreement for a given characteristic. In contrast, figure 5 for CCD and electronic supplementary material, figure S21 for DTW provide geometric visualizations of the specific subset of links, highlighting the strongest connections between parameters and emphasizing the most pronounced relationships. In particular, instead of visualizing all quantitative measures of agreement between all pairs of the considered characteristics and showing the measure values based on colour, the graph depicts only the pairs with values above a certain threshold, where the width of the edge represents the value. The graphs are presented in figure 5 (for the CCD metric) and electronic supplementary material, figure S22 (for the DTW metric) where the graph’s vertices represent characteristics and edges denote strong correlations which exceed the threshold 0.25. Similarly to the tree comparisons, the results between the cophenetic correlation and Baker’s Gamma methods agree fairly well, with some minor discrepancies. This supports the robustness of findings despite the chosen method. The cophenetic correlation and Baker’s Gamma estimates are also summarized in electronic supplementary material, tables S1−S4.

For the CCD metric, incidence was linked to various characteristics, including population health, VOCs genetic diversity, cross-country mobility and sociodemographic characteristics. Interestingly, among all the features, mortality was exclusively associated with population health, with no evidence of a connection between mortality and vaccination groupings and moderate correlation between incidence and vaccination groupings. VOCs genetic diversity grouping was linked to cross-country mobility data and sociodemographic data.

**Figure 6.**
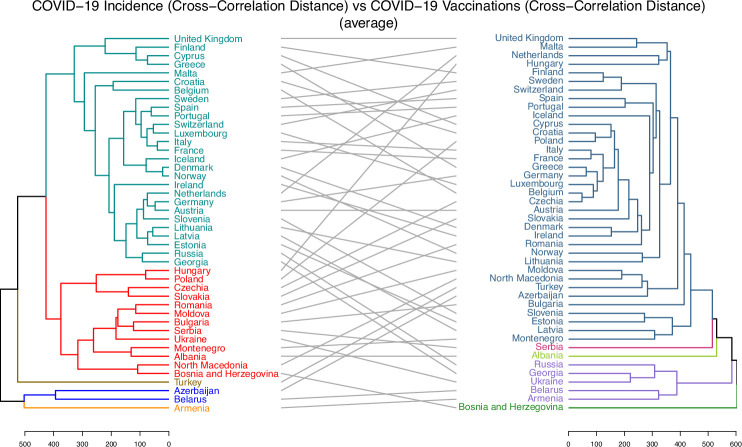
The visual alignment of the two hierarchical clustering trees for COVID-19 incidence (CCD) data (left tree) versus vaccination data (CCD) (right tree). A grey line connects the same country from each tree. Each tree has been split into *five* clusters for illustration purposes. The figure highlights the absence of cluster preservation. For instance, the violet cluster within the vaccination tree does not map to a single cluster of the incidence tree. The clusters of the incidence tree, to a significant degree, agree with geographical or cultural relationships, as will be discussed further.

**Figure 7.**
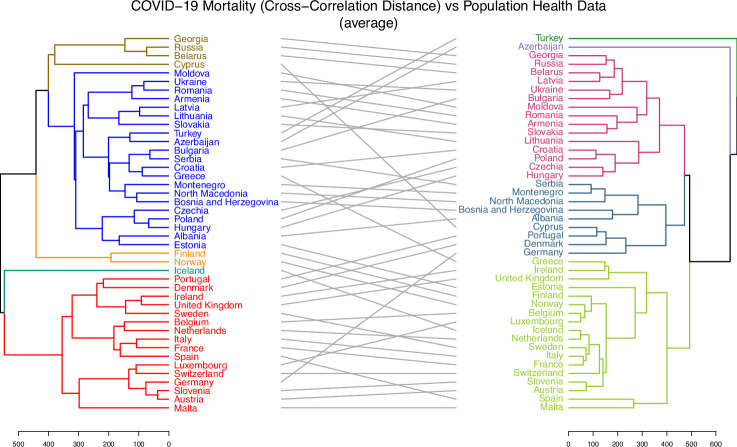
The visual alignment of the two hierarchical clustering trees for COVID-19 mortality (CCD) data (left tree) versus population health data (right tree). A grey line connects the same country from each tree. Each tree has been split into *five* clusters for illustration purposes. The figure highlights the agreement between the clusterings. For example, Sweden, Ireland and the United Kingdom are mapped from the red cluster of the mortality tree to the olive cluster on the population health tree. A significant portion of mappings are between clusters.

The examples of visual summaries of pairwise alignments of tree pairs for which cophenetic correlation and Baker’s Gamma statistics were computed are presented in figures 6 and 7 as well as electronic supplementary material, figures S24 and S25. Those summaries provide both the hierarchical trees for pairs of data sources as well as the corresponding visual links between the same countries located in those trees. Each of these figures displays a tree generated for one data type on the left and a corresponding tree for another data type on the right, with the same countries appearing in both hierarchies. Grey lines in the centre of each figure connect the same countries across the two trees. These figures were created for illustrative purposes, i.e. to visually highlight and emphasize how the hierarchies align by showing structural similarities between them. The choice to split each tree into five coloured clusters (using the k = 5 setting when specifying the pair of trees with the dendlist() function from the dendextend package) was arbitrary and made solely to facilitate easier visual interpretation of the agreement between the two hierarchies; it did not influence any part of the analysis. Each tree could have been shown in a single colour without affecting the analysis, but this might have made visual interpretation more difficult.

The stability of the findings using the leave-five-countries-out cross-validation approach is summarized in Supplemental Tables 7−10. The results were in agreement with the initial analysis based on the entire dataset of 42 countries. The sensitivity of the findings to the length of the aggregation interval is summarized in Supplemental Tables 11−14 for the 2-week interval (a twofold increase in length) and in Supplemental Tables 15−18 for the 4-week interval (a fourfold increase in length). The results remained stable across aggregation levels and were in agreement across different interval lengths.

## 4. Discussion

The COVID-19 pandemic has revealed unprecedented complexities that challenge traditional epidemiological modelling approaches. The dynamic nature of vaccination processes, nonlinear vaccination effects, viral evolution and heterogeneous spatio-temporal transmission patterns, modulated by varying NPIs, demand more sophisticated analytical frameworks. Our research highlights the importance of considering these complexities when evaluating the relationship between vaccination rates, mortality dynamics and incidence dynamics. The research on optimal long-horizon government policy, including NPIs and vaccination effects, remains still active even in 2025 [107–111]. Our methodological approach highlights potential relationships and underscores the need for further investigation into specific areas of the pandemic long-term dynamics.

*Q1: Is it generally true that countries with similar vaccination dynamics exhibit similar dynamics of mortality?*

Our clustering analysis of European countries, based on normalized mortality and incidence time series, captures pandemic-wide similarities, transcending wave-specific variations. We found that clusters based on mortality highly agreed only with clusters based on population health. This aligns well with existing evidence of certain chronic diseases amplifying the severity of COVID-19 [15]. However, this study did not find evidence that clusters based on vaccinations correlate with those based on COVID-19-related mortality. This result is not necessarily surprising: the reported infected individuals and COVID-19-specific deaths correspond to only a small fraction of the actual underlying counts.

Previous studies have shown that the relationship between COVID-19 incidence and mortality rates is complex, with various factors influencing these dynamics [112]. Early trends in incidence and mortality may not predict later outcomes due to the evolving nature of the virus and public health responses. Excess mortality can arise from chronic conditions exacerbated by COVID-19 [113], while increased testing may lead to better identification of cases without necessarily correlating with higher mortality rates [114]. There could be multiple explanations for this, including the acquisition of natural immunity. While it is impossible to determine this with certainty based on the available data, the previous studies indicate that natural immunity from recovered individuals is considered to be at least equivalent to vaccination protection [115], or even preferable for certain groups [116,117]. Studies have also linked higher COVID-19 vaccination rates with lower mortality rates [118], while heavily influenced by strain introductions on a different timeframe [119]. So our findings highlight the importance of understanding the complexity of the phenomena throughout of comparing normalized time series, derived by inference with natural immunity network effects, VOC introduction and NPI-timing effects.

*Q2: Is it generally true that countries with similar vaccination rates exhibit similar dynamics of incidence?*

In our study only a moderate correlation was observed between incidence and vaccination clustering. Incidence, unlike mortality, was linked with many factors, including public health, VOCs’ genetic diversity, cross-country movement and sociodemographics. The genetic diversity of VOCs was also linked to mobility and sociodemographics. In both instances, the association seems to be geographical rather than social. Indeed, this implies that patterns of mobility before the pandemic were preserved even after travel restrictions were implemented, potentially influencing the introduction dynamics of new phylogenetic lineages [14,120,121].

These findings are consistent with the notion that vaccines can shift symptomatic cases to asymptomatic cases, but may not substantially impact population susceptibility, at least at the demonstrated vaccine coverage speeds and COVID-19 vaccine effectiveness against evolving strains [122].

*Q3: Is it generally true that countries with outlier NPIs are outliers both globally and within their own spatially derived country groups?*

This study does not designate Belarus and Sweden, two European countries distinguished by their limited NPI measures, as extreme outliers, as might have been supposed at the beginning of the pandemic. Belarus could be deemed an outlier, not in isolation, but as part of small clusters of other post-Soviet nations.

The temporal dynamics of NPI implementation presents critical dimension, where early intervention timing may simply delay initial wave peaks rather than prevent them [119,123]. The duration and stringency of restrictions have varied significantly across regions, with European responses spanning diverse strategies, from minimal interventions (Sweden, Belarus) to adaptive approaches (Denmark) or sustained strict measures (Portugal, Spain, UK, Germany).

The influence of NPIs on the long-term incidence of COVID-19 has been a topic of debate, particularly regarding their effectiveness beyond immediate hospital load management. Several studies indicate that while NPIs were effective in reducing transmission rates during acute phases of the pandemic, their long-term impact on overall incidence appears limited due to delayed effects and the dynamics of epidemic resurgence [124,125]. This suggests that while NPIs can be crucial in controlling outbreaks initially, their role becomes less significant as the virus adapts and spreads more efficiently among populations [126], or leading to potential resurgences in cases after lifting restrictions [127].

These findings are in agreement with novel pandemic mitigation measures, which primarily highlight targeted approaches for specific population groups, prioritizing care and vaccination for identified target groups [128,129], and developing strategies for network-based NPIs [121,129–132], rather than relying on total or static NPIs. These findings also bring support for a novel approach to implementing dynamic NPIs proactively [133–137] and adapting to the epidemic phase [106].

### 4.1. Limitations of the study

It is important to emphasize that the identified associations are not necessarily causal. The secondary data did not come from a controlled experiment, and the studied associations may be influenced by a plethora of confounders. Additionally, the fact that the analysis used aggregated data counts may have affected the findings. The primary aim of this paper was not to directly prescribe or suggest policy adjustments, but rather to introduce a methodological approach capable of highlighting potential relationships and emphasizing the need for further investigation into specific areas. Our contribution lies in presenting a framework for analysing long-term pandemic data, including the disentanglement of complex effects arising from viral mutations (e.g. VOC strains) and vaccination campaigns. However, formulating explicit policy recommendations would require more in-depth analysis, particularly through individual-level modelling, which falls outside the scope of the present study.

The related concern for the analysed data is the under-reporting of epidemiological data [138], which is typical for respiratory diseases. In addition, there are differences in reporting and data quality between countries that can be due to differences in testing capabilities, reporting practices and policies, frequency and consistency of reporting, and population access to healthcare [96,139–141]. Consistency or comparability of reporting biases across countries is not assumed. It is recognized that each country has its own reporting system, accompanied by biases that may also vary over time. A method is proposed that identifies groupings of countries based on similarities in the dynamics of reported characteristics, without assumptions being made about the nature of those similarities. Reporting bias is considered one of the possible, and arguably one of the most significant, factors contributing to differences in these dynamics. The approach is designed to capture such influences.

The other concern is that the definition of a case may differ regionally. While lab-confirmed cases may seem straightforward, how cases are reported can differ. Cases may be ambiguously reported based on the day of the first symptom onset, the sample collection date or the sample processing date. Similar ambiguity exists for mortality; for example, an individual may die from COVID-19-related complications soon after formal recovery with negative lab tests. Therefore, reported incidence and mortality should only be treated as approximations for real-time series.

Non-epidemiological data are also subject to various shortcomings and biases. In particular, the mobility data were only available for the years 2011–2016, thus preceding the pre-pandemic period; moreover, Serbia and Montenegro were not included in these data [67,68]. Furthermore, aggregated statistics for the entire country’s sociodemographic, health and economic categories may not reflect fine details and particular subpopulations, thus potentially leading to *ecological fallacies* [142]. For instance, the population groups most affected by COVID-19 may have more limited access to the healthcare system than the ‘average’ population.

The analysed factors are not exhaustive, and many characteristics were not included in the present study and may be analysed in future studies. In particular, it would be interesting for future studies to explore the relations between various characteristics and excess mortality. Research in this area has already been conducted [143]. Besides providing an alternative source of information about COVID-related deaths, it may, for instance, also provide deeper insights into the effects of vaccination [144–146]. It should be noted, however, that the excess mortality data were not reported as frequently as other epidemiological data, with significant delays and different degrees of accessibility and availability for different countries [38]. Several potentially significant non-epidemiological factors were also left for future research. This includes secondary measures of NPI implementation [133,147], such as the stringency index [148], vitamin D deficiency [149], climate parameters [150], seasonal behavioural patterns [151], ethnic composition of the population [152], literacy [153], conformity to government regulations [130], political adherence [154], social and urban stratification [155–157], media influence [158], air pollution [159], diet [149], observed mobility [160,161] (e.g. Google Mobility Report, card transactions, cell phone records, carbon measurements of car and factory activities), founder and Matthew effects [162], age structure [163,164] and cross-reactive immunity from prior exposures [165]. As regards to the methodology, it also can be expanded by adding, for instance, other widely used tree comparison metrics, such as Fowlkes-Mallows index [166] or Robinson-Foulds distance [167].

It is also important to emphasize and further elaborate on the distinct advantages and limitations of CCD and DTW distances. This will help to clarify the contexts in which each metric may offer more meaningful insights, depending on the structure and characteristics of the studied time-series data.

More specifically, the DTW method is a well-established algorithm specifically designed to measure similarities between time series that may differ in speed. It accounts for both acceleration and deceleration, making it particularly well-suited for analysing time-series data. However, DTW has several important limitations. One of its key drawbacks is its high sensitivity to noise and outliers, which can be matched at corresponding time points and significantly distort similarity measurements [168]. This is especially critical in the context of disease reporting, where irregular, uneven or underreported data can affect the robustness of the results. Another limitation of DTW is that it is not invariant to scaling [169,170]. This necessitates normalization of the data. Although the data used in our analysis were normalized by population size, the population figures are not available for every reporting period. Instead, they are based on annual estimates recorded on 1 January of each year. This introduces potential bias into any population-based normalization of *per capita* incidence rates.

On the other hand, the CCD metric presents a different set of limitations. While its unitless nature allows for the comparison of time series across different spatial scales and data sources, it also has inherent drawbacks. Specifically, it may struggle with non-stationarity and non-linear dependencies [171], and it relies on linear shift assumptions rather than capturing more complex patterns [172].

It is also important to discuss the choice of the correlation cutoff value of 0.25, which was used to generate the connected graphs illustrated in figure 5 and electronic supplementary material, figure S22. During manuscript preparation, a sensitivity analysis was performed to evaluate different correlation cutoff values. The range of values considered spanned from 0.25 to 0.50, in increments of 0.05. Values above this range were not included, as at a threshold of 0.50 the graph began to fragment into disconnected components, failing to provide a connected or interpretable pattern.

The choice of the lower bound of 0.25 was informed by the literature, where values below this threshold are generally not recommended as meaningful for interpreting effect sizes [173,174]. When thresholds in the range of 0.25−0.30 were applied, the graph structure began to stabilize, making 0.25 a reasonable and effective choice for capturing potentially important links.

The choice of Euclidean distance on z-score-transformed variables was made because it represents the simplest and most interpretable approach. Mahalanobis distance was also briefly considered. While it is a valuable method that accounts for correlations between variables, it has several drawbacks that led us to exclude it from the present analysis. Specifically, Mahalanobis distance requires the computation and inversion of a covariance matrix, which can be computationally intensive and numerically unstable—particularly when the matrix includes highly collinear variables or is close to singular. Most importantly, in some cases, the covariance matrix may not be invertible due to singularity, making the method inapplicable for certain datasets. Our objective was to adopt a method that is robust and always broadly applicable across all realistic scenarios. In contrast, Euclidean distance offers simplicity and consistency, ensuring its applicability across diverse data types and configurations. This does not, however, preclude readers from using Mahalanobis distance as an alternative in cases where it is deemed appropriate, i.e. when a stable, non-singular correlation matrix is available for selected datasets, since the overall framework remains unchanged.

The other important potential limitation is that typically both incidence and mortality are not influenced or associated with individual single factors considered in the presented study individually, but rather by combinations of such factors, which may have potentially very complex interactions with both incidence and mortality and with each other [175,176] as well as influenced by the external factors such as cross-reactive immunity [165]. The study of all such interactions is not plausible, so the study goal was to identify the individual factors that may affect the studied outcomes for future, more scrutinized investigations.

It is also beneficial to discuss practical aspects of applying the method, such as potential implementation challenges, runtime considerations, software-specific dependencies and other possible limitations. The complete analysis code has been made publicly available on GitHub [95], ensuring transparency and reproducibility of the findings and making the methodology accessible to a broader audience.

Given the limited number of countries and time series, the computational requirements are minimal. The methodology can be executed on any operating system with R installed, and equivalent functionality can be implemented using Python libraries for time-series analysis and cross-correlation. Running the entire pipeline, including generating all outputs provided in the GitHub repository, takes less than an hour on a modern personal computer, highlighting the method’s practical applicability. The distance functions employed (e.g. CCD and DTW) are available natively or through standard R packages.

## 5. Conclusion

In conclusion, this study demonstrates the utility of clustering-based approaches in identifying patterns and relationships between COVID-19 dynamics and various public health and sociodemographic factors across 42 European countries. The results highlight distinct associations between different characteristics, with notable connections between vaccination, incidence, mortality, genetic diversity, mobility and sociodemographic factors. These findings offer valuable insights into the underlying drivers of epidemiological disparities, which can inform targeted public health interventions and policies tailored to specific regional and demographic contexts.

The method is particularly useful when primary characteristics (e.g. disease incidence, mortality rates) and numerous auxiliary variables are available, but their relevance is uncertain. It identifies key auxiliary variables by analysing clustering patterns, helping to prioritize further research. Although the analysis is associative, it supports targeted policy investigations into potential drivers of trends or the absence of effects across the entire multi-wave pandemic (e.g. beyond initial outbreaks).

## Data Availability

All data were obtained from public sources and were publicly available. The corresponding links for data sources have been provided in the Data folder on GitHub [95]. The entire analysis source code in R and in MATLAB has also been made publicly available on GitHub [95]. Supplementary material is available online [177].
